# Early Life Stress Detection Using Physiological Signals and Machine Learning Pipelines

**DOI:** 10.3390/biology12010091

**Published:** 2023-01-06

**Authors:** Zeinab Shahbazi, Yung-Cheol Byun

**Affiliations:** 1 Department of Mathematics Informatics, University of Barcelona, 08007 Barcelona, Spain; 2 Department of Computer Engineering, Major of Electronic Engineering, Jeju National University, Institute of Information Science & Technology, Jeju 63243, Republic of Korea

**Keywords:** early life stress, machine learning, physiological signals, prediction

## Abstract

**Simple Summary:**

Problem statement: Stress is one of the challenges of human life that in case of not curing may cause serious problems in human body. There are various type of stress that in this research we mainly focus of Early life Stress during the pregnancy. Aims and objectives: In this approach we are analyzing the stressed and relaxed categories of pregnant woman. Results: In this approach we have applied Machine Learning approach for the prediction and similarly due to data amount we have used oversampling approach for better comparison of the balanced and oversampled types.

**Abstract:**

Pregnancy and early childhood are two vulnerable times when immunological plasticity is at its peak and exposure to stress may substantially raise health risks. However, to separate the effects of adversity during vulnerable times of the lifetime from those across the entire lifespan, we require deeper phenotyping. Stress is one of the challenges which everyone can face with this issue. It is a type of feeling which contains mental pressure and comes from daily life matters. There are many research and investments regarding this problem to overcome or control this complication. Pregnancy is a susceptible period for the child and the mother taking stress can affect the child’s health after birth. The following matter can happen based on natural disasters, war, death or separation of parents, etc. Early Life Stress (ELS) has a connection with psychological development and metabolic and cardiovascular diseases. In the following research, the main focus is on Early Life Stress control during pregnancy of a healthy group of women that are at risk of future disease during their pregnancy. This study looked at the relationship between retrospective recollections of childhood or pregnancy hardship and inflammatory imbalance in a group of 53 low-income, ethnically diverse women who were seeking family-based trauma treatment after experiencing interpersonal violence. Machine learning Convolutional Neural Networks (CNNs) are applied for stress detection using short-term physiological signals in terms of non-linear and for a short term. The focus concepts are heart rate, and hand and foot galvanic skin response.

## 1. Introduction

Exposure to adversity is associated with a higher risk of negative health outcomes, such as early all-cause death, metabolic [1], mental, and neurodegenerative disorders [2,3]. Both the immune system and the central nervous system (CNS) are primarily “plastic” or susceptible to epigenetic changes in response to environmental cues during critical windows of development. Epigenetic changes caused by “biological embedding” [4] of stress can increase health risks in later life [5]. Although the flexibility of the brain, behavior, and immune systems is evident early in childhood, it persists into adulthood. We know relatively little about whether certain adult developmental phases are linked to increased vulnerability to adversity and long-lasting phenotypic alterations. Pregnancy may be a significant “developmental infection point”, during which the mother’s immune system experiences dramatic and continuing changes that are essential to both her and her unborn child’s survival [6]. Given that not all women who experience adversity throughout their lives suffer from ill health, more research is required to better understand the phenotypes and mechanisms linked to adversity exposure, considering any potentially sensitive times. Such data would enable the creation of novel diagnostic biomarkers, the detection of high-risk people, and improved disease targeting [7]. One crucial mechanism through which adversity exposure raises the risk of future disease is inflammation [8]. Pregnancy [9] and early childhood [10,11] are particularly vulnerable times for the immune system to change significantly, resulting in increased phenotypic plasticity and susceptibility to environmental stresses. Although higher pro-inflammatory proteins are frequently linked to trauma exposure, these biomarkers are not very specific, as they are also elevated in a wide range of physical and mental problems [12]. Additionally, because inflammation is multidimensional, using a single biomarker, such as C-Reactive Protein (CRP), is frequently insufficient to accurately identify people who are at risk [13,14]. Therefore, complicated, composite biomarkers have lately been requested by the National Institute of Health. Myeloid cells of the innate immune system, such as monocytes and macrophages, produce the most inflammatory cytokines. Therefore, this work aims to determine whether pro-inflammatory monocyte/macrophage phenotypes might reveal the distinct biological signature of adversity encountered throughout critical stages of immunological development.

A unique phenotypic marker of sensitive times should also adhere to a set of requirements for specificity concerning the timing of exposure and the genomic fingerprint. First, we reasoned that the M1/M2 phenotype shouldn’t be strongly related to overall adversity across the lifetime if it is unique to early life or pregnancy experiences. Second, sensitive periods shouldn’t be linked to alternative biomarkers if they are exclusively related to M1/M2. Therefore, we also examined levels of traditional pro-inflammatory and anti-inflammatory cytokines and C-Reactive Protein to see if they may offer similar insights (CRP). The endotoxin tolerance (ET), a different macrophage-associated feature that has been shown to be reliable in patients with sepsis across a wide age range, was compared to M1/M2 in a similar manner. After initial priming (such as by pathogen exposure), trained immunity and tolerance are “two opposed functional processes of innate immunity”, the former of which increases inflammatory reactivity while the latter decreases it. This epigenetic study is why we chose ET.

The main contribution of this paper is as below:

- An important approach in this research is on the healthy category of people that are at risk of stress during their pregnancy.
- Evaluating the early life stress based on the physiological signals using and verifying a multimodal CNN classifier based on Cont-RPs for stress classification.
- Utilizing the FGSR, HGSR, and HR signals, which are short-term (30 s or less) and have not been completely employed in other studies on stress classification.
- Focusing on non-linear physiological signals.

The following steps of this procedure are outlined in Section 2 along with a review of the most recent research in the field. The specifics of the suggested strategy and performance assessment are presented in Section 3. Section 4 includes information on the results and application, followed by the conclusion.

## 2. Related Work

The most recent developments in DF are covered in detail in this section. Two components are the primary focus. The first is the difficulties in digital forensics in blockchain, and the second is the forensic acquisition of social media content.

### 2.1. Early Childhood Pregnancy

Pregnancy, like early childhood, is a stage of life marked by high tissue growth rates, immunological tolerance development, and increased susceptibility to infection, which is a significant factor in mother and fetal mortality [15]. Contrary to appearances, immunosuppression, which helps control the inflammatory response, and chronic low-grade maternal inflammation are both characteristics of pregnancy [16]. Early in pregnancy, circulating blood monocytes penetrate the uterine lining and develop into “decidual macrophages”. Excessive inflammation at the maternal-fetal interface predicts premature birth and fetal death, which is linked to a pro-inflammatory “M1-like” macrophage phenotype [17,18]. Macrophages that express more “M2-like” genes contribute to the generation of immune-suppressive T-regulatory cells that inhibit rejection of the semi-allogenic (non-self) fetus (immunoregulatory and anti-inflammatory) [19]. These coordinated programs involving hundreds of genes and M1- and M2-like profiles show the influence of epigenetic processes in part. Inflammatory illnesses have been associated with a relative imbalance favoring M1-like expressed genes over M2-like expressed genes.

The primary cellular mediators of inflammation are macrophages. Inflammation is a complicated response to immediate and impending dangers [20], including coordinated transcriptional programs across hundreds of expressed genes. Therefore, only one or two serum protein indicators are insufficient to understand inflammation. Therefore, only one or two serum protein indicators are inadequate to understand inflammation. A pro-inflammatory M1/M2 polarization skew has been linked to an increased risk for various negative clinical outcomes, including atherosclerosis, diabetes, neurodegeneration, pregnancy problems, and treatment resistance for mental health [21]. Contrarily, a pro-inflammatory aggregate score of CRP, IL-6, TNF-a, and IL-1ß did not significantly correspond with adversity experienced during sensitive times. In the present investigation, only total lifetime adversity connected with the pro-inflammatory protein profile, and only when socioeconomic factors were considered, in contrast to prior studies that have linked early life adversity and CRP [22]. Notably, the average CRP level in this cohort was 2.5 mg/L (interval: 0.29–11.60), showing widespread low-grade inflammation associated with elevated cardiovascular disease risk [23]. The pro-inflammatory outcome’s favorable statistical distribution and the tiny (0.20) effect sizes may restrict the small sample size’s statistical power, but the M1/M2 phenotype may be a more accurate biomarker than serum proteins. Our findings might be influenced by the creation of new diagnostic biomarkers, experimental paradigms, and therapeutic strategies that revealed an unexpected discrepancy between two macrophage-associated behaviors. First, there was a correlation between more significant pro-inflammatory M1/M2 phenotypic skew and adversity during sensitive periods (but not overall life adversity). Second, it has been proven that sepsis in adults and children is related to diminished expression of an immunosuppressive, endotoxin-tolerant phenotype across the lifespan [24,25,26]. Furthermore, this connection remained substantial even after accounting for exposure during a sensitive period.

### 2.2. Stress Prediction of Drivers

Various diseases and reduced work efficiency can be caused by excessive mental stress [27,28,29,30]. The relationship between driving safety and stress is particularly apparent in driving situations. Among the factors contributing to traffic accidents is stress that impairs driving performance or reduces the ability to make decisions in dangerous situations [31,32]. Thus, several studies have attempted to reduce traffic accidents by recognizing stress early [33,34,35]. Various measurements have been used to detect drivers’ stress, categorized into vehicle motion, facial behavior, and physiological measurements. In addition to measuring how fast drivers accelerate and brake, lane position, steering angle, and handle movement patterns [36,37,38], vehicle motion measurements are also considered. The types of vehicles, driving habits, and road conditions can all affect the results of such measures. Similarly, measuring facial behavior is possible without interfering with the driver, such as looking straight ahead, dilation of the pupil, blink rate, yawning, and head movement. Depending on the conditions, these measurements are not reliable under poor lighting conditions, bad weather, at night, or when a driver is wearing eyeglasses. Lighting conditions or driver behavior do not affect physiological measurements unrelated to stress. As an additional benefit, physiological signals collected by bodywear can provide valuable insights into a driver’s internal state, thus allowing for stress recognition [39,40]. Since autonomic nervous system activity is associated with stress response, galvanic skin response (GSR) and heart rate (HR) are often used as reliable stress indicators. Therefore, stress recognition problems can be solved by utilizing a variety of physiological signals obtainable at low cost and with a wide availability of equipment [41,42,43]. Physiological signals have been extracted as features primarily in the time or frequency domains in studies of stress recognition. Window sliding strategies are typically used to truncate time series segments into time domain features, whereas low- and/or high-frequency regions extract frequency domain features [44,45]. These features were used to calculate and differentiate between stressed and non-stressed conditions using statistical measures like mean, standard deviation, skewness, and kurtosis. Additionally, other studies [46,47] employed domain-dependent features defined by experts with specific knowledge about signal types or human mental stress. These features are effective in certain situations but are not robust under certain conditions, for example, when noise or intrapersonal variability is present. Table 1 shows the different types of features for stress recognition based on physiological signals.

### 2.3. Stress and Anxiety

Virtual reality exposure treatment (VRET) can be extremely effective in identifying and perhaps treating a variety of anxiety problems. The ability of a psychologist to engage with 3-dimensional settings and alter treatment situations in accordance with the demands of each client is among the key benefits of VRET systems. The client’s anxiety state must be monitored across the VRET session, though, in order to achieve this effectively. Consequently, a mental stress detection system is required to properly utilize the benefits offered by the VRET system. Wearable biofeedback sensors can gather the client’s physiological data. The categorization models for anxiety levels might be trained using signals like skin temperature, galvanic skin response (GSR), and blood volume pressure (BVP). The various advantages of this type of VRET system are highlighted in this research [45], as they integrate VRET with mental stress detection. They describe and demonstrate a methodology for recognizing anxiety levels that is a component of our created virtualized VRET system. Thirty individuals’ biological signals were monitored throughout VRET-based therapy for fear of public speaking. The collected data were utilized to train a four-level anxiety recognition model, with the terms “low”, “mild, “moderate”, and “high” denoting the severity of the anxiety instead of distinct anxiety disorder groups. With the signal fusion-based support vector machine (SVM) classifier, they obtained cross-subject prediction performance of 80.1% (assuming leave-one-subject-out cross-validation) and 86.3% (utilizing 10 × 10-fold cross-validation). Human stress recognition is being applied in a range of situations, which as stress brought on by financial strain, psychological strain, trauma, and medical problem. To replicate the actual conflict situation, ref [49] developed a virtual reality (VR) technology-based digital battlefield driving scenario and replicated stray gunshot impulses within the same. To study the pressure situation brought on by the bullet sensations, the VR scene is then integrated with real-time physiological sensors, such as electrocardiography (ECG), a skin conductance (GSR) sensor, and an eye-tracking device.

## 3. Proposed Early Life Stress Detection

In the current cross-sectional investigation, low-income women from various ethnic backgrounds who had experienced various types of interpersonal trauma were examined to better understand how social adversity predicted immunological phenotypes. For the following reasons, we focus on monocyte/macrophage phenotypes:

- Demonstrate developmental sensitivity during pregnancy and the first several years of life.
- Promotes inflammation-related illness.
- Assist in maintaining the proper balance between innate and adaptive immunity, which has an impact on immunosuppression.

**Figure 1:**
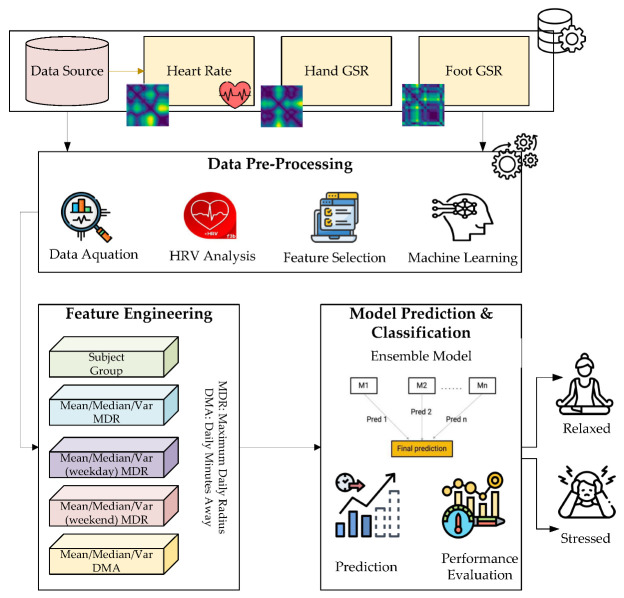
Overview of the proposed framework.

Our major prediction was that more adversity would be positively connected with changes in a macrophage-associated M1/M2 RNA profile due to a relative increase of pro-inflammatory over anti-inflammatory and immunoregulatory genes. The early years (here defined as being from birth to age five) and the first trimester of pregnancy were these two vulnerable times in a woman’s life. Figure 1 presents the proposed early life stress prediction details.

### 3.1. Dataset

The Child-Parent Psychotherapy Health Investigation (CPP-HEALTH), conducted between 2013 and 2015, provided the data for the current study. 62 women and their kids who wanted treatment for their child’s exposure to interpersonal trauma were registered by CPP-HEALTH. Examining biological markers of trauma and treatment-related adjustments in psychological functioning after involvement in a previously approved dyadic intervention, child-parent psychotherapy, was the aim of this study [50]. The inclusion criteria included being a native speaker of either English or Spanish and having biological moms with children between the ages of 2 and 6 who had experienced trauma. Homelessness, ongoing domestic abuse, substance addiction, having a child who is in a state ward, being pregnant right now, a child developmental issue, psychosis, or long-term illnesses for either the mother or the child were all considered exclusions. Participants who were mothers gave written consent and received payment. The Institutional Review Boards of Zuckerberg San Francisco General and the University of California San Francisco approved the study. The sample with serum cytokine data has n = 53, while the subsample of these 53 women having gene expression data has n = 42. Participants with a high school diploma received a code of 0 for fewer than 12 years of attendance and several 1 for 12 or more. The 2016 Census criteria were used to calculate poverty (see “Methods” section). The total score on the PTSD Symptom Scale Interview, Center for Epidemiologic Studies-R CESD Total score on the depression scale.

### 3.2. Oversampling Approach

Oversampling is one of the techniques to increase the number of the dataset. This method can be useful for ML methods where the model fit can be influenced by several duplicate samples for a particular class and if the distribution is skewed. This could involve iteratively learning coefficients-based techniques like stochastic gradient descent-based artificial neural networks. Support vector machines and decision trees are two examples of models that may be impacted. In this scenario, the number of datasets is less and to be able to achieve better training results, we have applied oversampling approach to our dataset and increased the amount of data to n = 120 for serum cytokine and n = 108 for gene expression data.

### 3.3. Characteristic of Participants

In this study, 53 mother-child pairs participated in the current investigation and gave blood samples for inflammatory cytokine tests and baseline assessments Table 2. A convenient subset of 42 was found to have gene expression phenotypes. According to power analyses, a moderate effect size (d = 0.40) at n = 44 may be detected with 80% power. Women were mostly non-white Hispanic (72%), 32 years old on average (range: 22–51 years), and lived in poverty (66%; Table 2). The average time since the difficulty mentioned in the pregnancies documented here was 4 years (range: 2–6 years). Total life adversity, or TLA, is the number of adverse events participants reported experiencing on average over the course of their lives. Of these, early life adversity, or ELA, was reported by 46% of participants, and pregnancy adversity, or PA, by 66% of participants, indicating high but variable levels of trauma exposure at different developmental stages. There was no discernible correlation between ELA and PA. At baseline, individuals had a mean CRP level of 2.50 (SEM: 0.42, Range: 1.50–3.50), were overweight, had higher PTSD and depressive symptoms, and had low-grade chronic inflammation.

### 3.4. Classification and Feature Extraction

**Figure 2:**
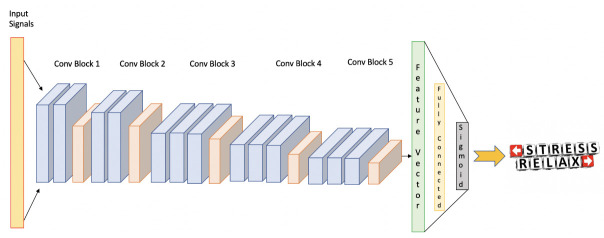
Overview of the CNN framework.

We built a multidimensional CNN model, comprised of the three physiological parameters and one dense layer to develop the probability values of each stress category from the output signals. This allowed us to automatically learn the stress-relevant features of the three types of physiological signals. The Cont-RPs of each type of signal were provided as inputs for each CNN during training. To acquire the inherent features present in the Cont-RPs of the FGSR, HGSR, and HR signals, three CNNs have been used. The process is broken down into three different stages: first, starting to learn reflective feature space using the multiple CNNs through the Cont-RPs of the three frequency varieties; second, incorporating their symmetrical outputs into one incorporated recognition variable; and third, creating class probabilities based on the representation vectors by learning the relationships between them using a fully-connected layer including a nonlinear function. Although our CNNs feature the same structure as the initial portion of the VGG16 model [51], many of the parameters were trained from scratch using the training dataset. Figure 2 shows the overview of the presented CNN framework. To put it another way, each CNN was made up of five convolution blocks, each of which had a two-dimensional inversion, a rectified linear unit (ReLU) activation, and a max pooling layer. Two fully connected layers were present in the first two convolution blocks (ConvBlocks 1 and 2), however, three convolution layers were added to the subsequent blocks. Additionally, all fully connected layers were processed using 3 × 3 filters with a stride of 1, and the max pooling layer was processed using 2 × 2 filters with a stride of two. While pooling layers minimize the spatial component of extracted features, convolution layers learn spatial information and extract features using kernels with sliding window strategies. The resulting three feature maps were flattened (i.e., three vectors with a dimension of 256) and concatenated within one consolidated depiction vector with a size of 768 after passing along through the fifth convolution block in each CNN. Each variable was then supplied to the dense layer, as well as the output layer’s perceptron was the nonlinear function, which was used to forecast the probabilities of the two classes (the stressful and relaxing states).

## 4. Results

### 4.1. Experimental Setup

There were fewer samples of the relaxed state (minority class) in the defined dataset than those of the stressed state (majority class). In contrast to the minority class samples, which were poorly learned, the classifier derived from the unbalanced data was susceptible to bias toward the majority class. To address the class imbalance issue, we randomly undersampled the stressed samples to equalize the class distribution between the stressful and relaxed stages. As a result, we collected 5348 10 s samples and 1872 30 s samples for the model development. We employed recording-wise cross-validation with a leave-one-recording-out approach to evaluate the efficacy of the suggested approach. In other words, we used eight recordings for model training and one for testing in each cycle. This approach was carried out to assess the trained model’s generalizability for new recordings of people who weren’t in the training set. We used a grid search to find the ideal hyperparameters, such as the learning rate, batch size, and the number of epochs. As an illustration, we looked at a range of different batch sizes from 1 to 20, epoch counts from 1 to 30, and learning rates from 0.1 to 0.00001. Then, using a batch size of 4, we trained our multimodal CNN model for 15 epochs. We then employed the Stochastic Gradient Descent (SGD) optimizer for the weight update with a learning rate of 0.001. Using the F1-score, recall (sensitivity), accuracy, precision (positive predictive values), and area under the curve, we evaluated classification performance (AUC). Precision, recall, and the F1-score calculated for each class show a method’s ability to distinguish between different classes, whereas accuracy and the AUC characterize the overall performance over all data types. Calculations for these indexes are as follows:

(1) Accuracy=(XP+XN)/(XP+XN+YP+YN)∗100

(2) Precision=XP/(XP+YP)∗100

(3) Recall=XP/(XP+YN)∗100

(4) F1−Score=(2∗Precision∗Recall)/(Precision+Recall)

where *XP* represents the number of positively classed samples that were correctly identified, *XN* represents the number of negatively identified samples that were incorrectly identified, and *YP* represents the number of negatively identified samples that were mistakenly identified as positive. *YN* is the number of favorable models that were mistakenly categorized as unfavorable.

### 4.2. Statistical Analysis

Inflammatory cytokines were blom-transformed, and gene counts were log2 normalized. All variables were checked for abnormalities. Each adversity component was entered into multivariable regression models as a predictor of the M1/M2 phenotype with and without adjustment for two different sets of covariates. Age, BMI, antidepressant use, and Hispanic ethnicity were among the demographic and medical factors in Model 1 that were highly representative of our group and were substantially linked to inflammatory cytokines in the literature [52]. Due to the small number of participants—four—we could not include breastfeeding as a covariate. Model 2 contains socioeconomic status variables such as family poverty defined by the census, US birthplace, and education. Similar specificity studies for IP, pro-inflammatory (PRO), and anti-inflammatory (ANTI) cytokines were performed. To determine whether certain adversity variables showed relationships with immunophenotypes that were distinct from one another and mental symptoms, we performed multivariable regression analyses.

### 4.3. Immune Biomarkers Adversity Associations

**Figure 3:**
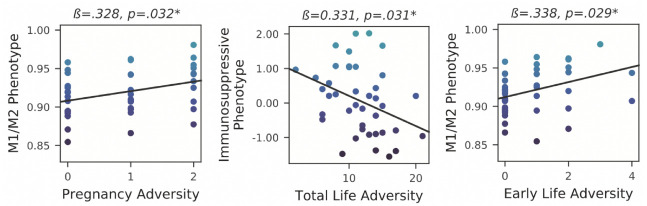
Depending on when the exposure occurred, different immunological phenotypes are linked to hardship in life. * for *p* < 0.05.

A greater M1/M2 RNA score was substantially linked with ELA and PA, but not with TLA (all *p*’s < 0.05). Figure 3. On the other hand, the endotoxin intolerance (ET) immunosuppressive phenotype was strongly correlated with a lower score when TLA levels were higher exclusively (rather than ELA or PA).

Contrarily, in no investigation did ELA or PA show a significant relationship with serum pro-inflammatory cytokines (PRO) in Table 3.

Furthermore, despite adjusting for socioeconomic factors including income (which also had a substantial independent positive correlation with TLA), growing up in the US, and training, TLA was only significantly related to PRO (both ns). Higher PA, but not ELA or TLA, was significantly associated with decreased anti-inflammatory cytokines (ANTI) in unadjusted analyses; however, after age, BMI, antidepressants, and ethnicity were taken into consideration, the association ceased to be significant.

The *p*-value, commonly known as a probability level, is indeed a numerical indicator of where and how probable to believe that the facts you have are the result of random chance (i.e., that the null hypothesis is true). The *p*-value, which ranges from 0 to 1, is frequently used to indicate the statistical relevance level. The evidence that you should reject the null hypothesis is greater the *p*-value is smaller. ELA continued to be substantially correlated with the M1/M2 RNA score in multivariate analysis Table 4, independent of PA, PTSD, or depressive symptoms (all *p*’s < 0.05). Notably, the connection of PA with M1/M2 displayed a trend that was not statistically significant when ELA was present (*p* = 0.052).

Similar to this, the TLA-ET connection persisted after accounting for ELA and PA’s potential confounding effects (*p* = 0.031), as well as PTSD or depressive symptoms (*p* = 0.026) Table 5. Toll-like receptor 2 (TLR2) demonstrated the strongest Pearson correlation with both ELA and PA of the genes making up the M1/M2 phenotype, according to exploratory posthoc tests for hypothesis generation (r’s > 0.20; Figure 3. They were exploratory, hence multiple comparisons were not corrected.

### 4.4. Performance Evaluation

We tested the effectiveness of the suggested stress detection method using input signals that were 10 s and 30 s in length. Table 6 provides a comprehensive overview of the classification findings. According to this table, the overall classification accuracy (and F1-score) for the 30 s input signals was 95.69%, compared to 92.35% for the 10 s input signals. Furthermore, with AUC values of 0.9872 and 0.9621, respectively, the AUC difference between the 30 s and 10 s input signals was not especially large.

The results are shown in Table 7 after we contrasted the performance of our multimodal CNN model with that of three unimodal CNNs for the FGSR, HGSR, and HR signals. Using all three physiological signals together resulted in a considerable improvement in classification performance, ranging from 2 to 36 percent for 10 s signals and from 5 to 33 percent for 30 s signals. Additionally, the FGSR-CNN of the three unimodal CNNs showed a reasonably decent performance, whereas the HR-CNN showed a somewhat subpar performance. The HGSR-accuracy CNNs lost 10 to 14 percent of their accuracy compared to the FGSR-CNN, depending on the length of the input signals. There was little change in the model performance’s variability between 30 s and 10 s signals in the FGSR. In other words, the length of the input signal had no discernible impact on the effectiveness of stress detection using FGSR. Since they are believed to represent characteristics that can distinguish between stressed and relaxed states, the Cont-RPs of short-term FGSR signals may be helpful for stress classification. In comparison to the long-term HGSR signal, the short-term HGSR signal performs only marginally better for 10 s signals than for 30 s signals, suggesting that it may be a more accurate indicator for stress detection.

Furthermore, we evaluated the classification performance of the multimodal CNN in comparison to that of two basic neural network models. All three models in Table 8 used CNN architecture to perform stress classification and extract features from FGSR, HGSR, and HR signals. To do this, a multimodal CNN was created by concatenating three vectors and entirely linked layers. Using three one-dimensional FGSR, HGSR, and HR sequences, the multimodal 1-D CNN classifier determines the likelihood of stressed and relaxed states. The three inputs are the preprocessed signals before generating Cont-RPs. The suggested multimodal CNN has the same structure as the multimodal VGG16 model, however, it employs the default parameters of the existing VGG16 without parameter learning. According to Table 8, the performance of two models (multimodal CNN and multimodal VGG16) with three Cont-RPs as input is around 1 to 12 percent and 1 to 9 percent better than that of the multimodal 1-D CNN model with the one-dimensional sequence as input for 30 s and 10 s input signals, respectively. According to the findings, one-dimensional signals—which were the predominant method employed in earlier studies—have less favorable characteristics for stress classification. The multimodal CNN model performs 8 to 10 percent better than the multimodal VGG16 model, while having the same VGG structure. It has acquired this because Cont-RP samples have taught it stress-relevant features.

According to Table 8, the comparison between different ML algorithms is provided using the oversampling approach. In this process, the applied algorithms focus on two categories of stressed and relaxed people. Oversampling approach was used in this step due to the little amount of data, with the goal of increasing the amount of data to have the comparison of other algorithms and thus improve system performance.

## 5. Conclusions

At crucial ages of childhood and adult development, exposure to psychosocial adversity has a significant impact on health over the course of a lifetime. But the mechanisms by which adversity “biologically embeds” itself are still unknown, and not everyone who is exposed faces the same risk. For the sake of Precision Medicine care, screening methods must be improved so that they incorporate an assessment of issues via a developmental lens and usable biomarker panels. In this study, a biomarker for poor pregnancy outcomes and early life adverse events (ELA), the macrophage-associated M1/M2 phenotype, is identified (PA). Even after controlling for sociodemographic, physiological, and psychological variables, we discovered that higher ELA and higher PA were linked to a greater relative expression of M1 vs M2-like genes in CD14+ circulating monocytes, indicating a pro-inflammatory imbalance. It is suggested that M1 specificity M2 is a biomarker of adversity experienced during these two sensitive periods by the absence of a significant connection between the M1/M2 phenotype and total lifetime adversity (TLA) exposure. According to new studies, a woman’s pregnancy is both a crucial time for imbuing her immune system with experience and a “vessel” for the fetus. A healthy balance between maternal M1 and M2 decidual macrophage phenotypes in the uterine lining is generally recognized as being necessary for a successful pregnancy, from implantation to fetal tolerance to birth. Due to the small quantity of data available, we compared oversampling and without sampling in this study to assess the suggested strategy from various perspectives and apply various machine learning algorithms to two aspects of stressed and relaxed pregnant cases.

## 6. Limitations and Strength

These statistics came from a sample of multicultural, low-income women who experienced a lot of violence and other hardships; therefore, conclusions may not apply to men or different populations of women. Due to clinic-based recruitment as a restriction, women were only questioned about difficulty experienced during one specific pregnancy; hence, any potential additional adversity during later pregnancies was not evaluated. The evidence indicates that monocyte/macrophages are likely to be most developmentally sensitive during early life and pregnancy, but additional acute phases of development may be relevant. We accept that in vivo macrophage phenotypes can differ from overly simplistic in vitro frameworks of the M1/M2 spectrum because they are dynamic, multimodal, and tissue-specific.

## 7. Future Study

Future research should combine cell surface and functional phenotyping with gene expression phenotyping. To determine whether M1 and M2 expression is co-expressed on single cells or signifies a change in underlying myeloid lineage cell subsets, single-cell RNA seq would be required. Adversity exposure is widely recognized as a risk factor for chronic disease on par with unhealthy eating habits and lack of exercise. However, little is known about the immunological mechanisms underlying this association. The M1/M2 RNA phenotype is a novel and distinct adulthood biomarker of adversity exposure across two critical developmental phases in the female lifetime. Our study indicates directions for developing translational research aimed at these mechanisms. With the help of advanced biomarker panels and developmentally targeted adversity screening, such insights may advance Precision Medicine and help people and prevent adversity-related morbidity.

## Figures and Tables

**Table 1:** State-of-the-art for feature types in stress recognition using physiological signals.

Domain Features	Physiological Signals	Examples of Features
Time	ECG, BVP, BR, HR, GSR, SpO2, ST	- Number of peaks - Adjacent elements means between various elements - Rise time - Mean - Median - Sum -RMS - Skewness - Kurtosis - Amplitude - Max & Min - Interquartile range - SD
Nonlinear [48]	ECG	- RQA - RP - Poincare plot
Frequency	ECG, RSP, GSR	- Power sum - Entropy - Power Spectrum density - The average power of LF/HF ratio - Spectral peak features
Domain Dependent	ECG, RSP, EMG, GSR	- Feature generation based on trends - Mean/Variant HP - GSR variation - RMS and SDCC between product - GSR variation & interpolation of first order

**Table 2:** Characteristics of the sample’s sociodemographic, adversity exposure, mental health, and physical health.

Samples of Characteristics	n = 53	n = 42
Symptoms of depressive (CESD-R)	24.98	24.60
Body mass index	27.57	27.53
Pregnancy adversity	0.91	0.88
Current antidepressant use	6	4
Age of mother	32.10	31.84
Age of child	50.38	49.53
Poverty of family	35	29
Diversity of early life	0.81	0.81

**Table 3:** One inflammatory marker was chosen as the outcome in each linear regression model, and the chosen life adversity component was chosen as the independent variable. The average blom-standardized scores for IL-1RA, IL-4, IL-10, and IL-1B, IL-6, TNF-a, and CRP are included in the anti-inflammatory and pro-inflammatory aggregates, respectively. Definitions of phenotypes are provided in the procedures. Age, BMI, Hispanic ethnicity, and current antidepressant use were factors in the adjusted model 1; high school education, family poverty, and US birthplace were covariates in the adjusted model 2. Data for three people were lacking when it came to overall life hardship.

Adversity Exposure Tunning	Phenotype of M1/M2		
	β	df	*p*
**Pregnancy**			
Unadjusted	0.332	2.226 (36)	0.032
M1 adjusted	0.404	2.764 (36)	0.009
M2 adjusted	0.324	2.049 (37)	0.048
**Earlylife**			
Unadjusted	0.337	2.265 (40)	0.029
M1 adjusted	0.308	2.094 (36)	0.043
M2 adjusted	0.311	2.114 (37)	0.042
**General Lifespan**			
Unadjusted	0.173	1.025 (38)	0.420
M1 adjusted	0.054	0.315 (34)	0.755
M2 adjusted	0.051	0.301 (35)	0.765

**Table 4:** Adversity at vulnerable times and mental health symptoms have both single-factor and multiple-factor associations with the M1/M2 phenotype.

#	M1/M2 Phenotype		
	β	*t*-Test	*p*-Value
**State of Mind**			
PTST (PSSI (df = 40))	0.180	1.157	0.254
Depression	0.074	0.470	0.641
**M1 df = 39**			
Pregnancy adversity	0.291	2.008	0.052
Difficulties of Early life	0.320	2.160	0.050
**M2 diversity + state of mind**			
pregnancy adversity	0.312	1.916	0.063
Difficulties of Early life	0.440	2.282	0.027
PTSD (PSSI)	0.213	0.627	0.470
Depression (CESD-R)	−0.155	−1.052	0.208

**Table 5:** Adversity throughout life, adversity during vulnerable times, and mental health both independently and jointly correlate with the endotoxin tolerance phenotype.

#	Phenotype of Endotoxin Tolerance		
	β	*t*-Test	*p*-Value
**State of Mind**			
PTST (PSSI (df = 40))	0.115	0.730	0.470
Depression (df = 40)	0.291	1.327	0.342
**M1 Multivariate (df = 36)**			
Pregnancy Adversity	0.213	1.375	0.178
Difficulties of Early life	0.229	0.852	0.575
General life difficulties	−0.442	−2.607	0.031
**M2 Multivariate (df = 36)**			
Depression (CESD-R)	1.182	0.805	0.426
PTSD (PSSI)	0.034	0.143	0.426
General life difficulties	−0.485	−2.432	0.037

**Table 6:** Performance of the suggested method’s classification depending on input signal length.

Length of Input	Classes	Precision	Recall	F1	Accuracy	AUC
30 s	Relaxed	96.1%	96%	95.9%		
		95.91%	95.69%	95.69%	95.69%	0.9872
	Stressed	95.9%	96.02%	96%		
10 s	Relaxed	92.6%	91.9%	92.1%		
		91.69%	92.80%	92.35%	92.35%	0.9621
	Stress	91.9%	93%	92.5%		

**Table 7:** Performance of the suggested method’s classification depending on input signal duration and sensor type.

Signal Length	Input Type	Stressed		Relaxed		Overall Accuracy
		P	R	P	R	AUC
30 s	HR	67.47%	59.97%	64.47%	66.02%	0.6296
	HGSR	82.93%	79.70%	82.98%	77.02%	0.7847
	FGSR	92.89%	87.72%	89.89%	92.72%	0.9093
	3 types	95.89%	98.02%	95.91%	95.90%	0.9882
10 s	HR	63.98%	61.98%	55.79%	57.65%	0.5985
	HGSR	83.78%	82.89%	83.79%	79.02%	0.5985
	FGSR	92.90%	88.72%	89.85%	92.60%	0.9123
	3 types	91.9%	93.00%	92.6%	91.9%	0.9621

**Table 8:** Comparison between the results of over-sampling and without-sampling using original data).

Without Sampling							
**Length of** **Signal**	**Input** **Type**	**Model for** **Classification**	**Stressed**		**Relaxed**		**Total** **Accuracy**
			**P**	**R**	**P**	**R**	
30 s	Cont-RP	Multimodal CNN	95.89%	96.02%	96.12%	96.00%	95.89%
	1-D sequence	Multimodal 1-D CNN	81.78%	85.81%	86.91%	81.22%	82.55%
	Cont-RP	Multimodal VGG16	88.00%	82.00%	85.33%	86.22%	84.22%
10 s	Cont-RP	Multimodal CNN	91.09%	93.00%	92.06%	92.00%	92.44%
	1-D sequence	Multimodal 1-D CNN	82.22%	83.44%	85.44%	81.55%	82.44%
	Cont-RP	Multimodal VGG16	83.66%	80.44%	83.66%	85.55%	84.00%
Oversampling							
30 s	Multimodal CNN		96.90%	97.20%	97.40%	97.85%	97.98%
	XGBoost		96.70%	96.93%	97.15%	97.00%	97.02%
	Random Forest		95.60%	96.01%	96.00%	95.30%	95.83%
	SVM		82.10%	82.93%	84.40%	83.90%	83.01%
	Logistic Regression		80.03%	81.00%	82.25%	81.50%	81.20%
10 s	Multimodal CNN		93.08%	94.05%	94.02%	93.09%	94.01%
	XGBoost		92.03%	91.07%	92.08%	91.09%	92.05%
	Random Forest		91.08%	92.00%	92.04%	91.09%	92.00%
	SVM		79.07%	79.01%	79.09%	80.03%	80.00%
	Logistic Regression		76.08%	77.03%	77.06%	77.09%	77.05%

## Data Availability

Not applicable.
